# Establishment of Intestinal Organoid from *Rousettus leschenaultii* and the Susceptibility to Bat-Associated Viruses, SARS-CoV-2 and Pteropine Orthoreovirus

**DOI:** 10.3390/ijms221910763

**Published:** 2021-10-05

**Authors:** Mohamed Elbadawy, Yuki Kato, Nagisa Saito, Kimika Hayashi, Amira Abugomaa, Mio Kobayashi, Toshinori Yoshida, Makoto Shibutani, Masahiro Kaneda, Hideyuki Yamawaki, Tetsuya Mizutani, Chang-Kweng Lim, Masayuki Saijo, Kazuaki Sasaki, Tatsuya Usui, Tsutomu Omatsu

**Affiliations:** 1 Laboratory of Veterinary Pharmacology, Department of Veterinary Medicine, Faculty of Agriculture, Tokyo University of Agriculture and Technology, 3-5-8 Saiwai-cho, Fuchu, Tokyo 183-8509, Japan; Mohamed.elbadawy@fvtm.bu.edu.eg (M.E.); s156174y@st.go.tuat.ac.jp (K.H.); s193249s@st.go.tuat.ac.jp (A.A.); skazuaki@cc.tuat.ac.jp (K.S.); 2 Department of Pharmacology, Faculty of Veterinary Medicine, Benha University, Moshtohor, Toukh 13736, Elqaliobiya, Egypt; 3 Center for Infectious Diseases of Epidemiology and Prevention Research, Tokyo University of Agriculture and Technology, 3-5-8 Saiwai-cho, Fuchu, Tokyo 183-8509, Japan; s163602q@st.go.tuat.ac.jp (Y.K.); s176469x@st.go.tuat.ac.jp (N.S.); tmizutan@cc.tuat.ac.jp (T.M.); 4 Faculty of Veterinary Medicine, Mansoura University, Mansoura 35516, Dakahliya, Egypt; 5 Laboratory of Veterinary Pathology, Department of Veterinary Medicine, Faculty of Agriculture, Tokyo University of Agriculture and Technology, 3-5-8 Saiwai-cho, Fuchu, Tokyo 183-8509, Japan; s212838s@st.go.tuat.ac.jp (M.K.); yoshida7@cc.tuat.ac.jp (T.Y.); mshibuta@cc.tuat.ac.jp (M.S.); 6 Laboratory of Veterinary Anatomy, Department of Veterinary Medicine, Faculty of Agriculture, Tokyo University of Agriculture and Technology, 3-5-8 Saiwai-cho, Fuchu, Tokyo 183-8509, Japan; kanedam@cc.tuat.ac.jp; 7 Laboratory of Veterinary Pharmacology, School of Veterinary Medicine, Kitasato University, 35-1, Higashi 23 Ban-cho, Towada, Aomori 034-8628, Japan; yamawaki@vmas.kitasato-u.ac.jp; 8 Department of Virology I, National Institute of Infectious Diseases, Toyama 1-23-1, Shinjuku, Tokyo 162-8640, Japan; ck@nih.go.jp (C.-K.L.); msaijo@nih.go.jp (M.S.)

**Keywords:** bat, natural host, organoid, long-term stable culture, virus susceptibility, SARS-CoV-2, ACE2, TMPRSS2

## Abstract

Various pathogens, such as Ebola virus, Marburg virus, Nipah virus, Hendra virus, Severe Acute Respiratory Syndrome Coronavirus (SARS-CoV), Middle East Respiratory Syndrome Coronavirus (MERS-CoV), and SARS-CoV-2, are threatening human health worldwide. The natural hosts of these pathogens are thought to be bats. The rousette bat, a megabat, is thought to be a natural reservoir of filoviruses, including Ebola and Marburg viruses. Additionally, the rousette bat showed a transient infection in the experimental inoculation of SARS-CoV-2. In the current study, we established and characterized intestinal organoids from Leschenault’s rousette, *Rousettus leschenaultii*. The established organoids successfully recapitulated the characteristics of intestinal epithelial structure and morphology, and the appropriate supplements necessary for long-term stable culture were identified. The organoid showed susceptibility to Pteropine orthoreovirus (PRV) but not to SARS-CoV-2 in experimental inoculation. This is the first report of the establishment of an expandable organoid culture system of the rousette bat intestinal organoid and its sensitivity to bat-associated viruses, PRV and SARS-CoV-2. This organoid is a useful tool for the elucidation of tolerance mechanisms of the emerging rousette bat-associated viruses such as Ebola and Marburg virus.

## 1. Introduction

Bats are one of the most important natural reservoirs for a variety of emerging viruses that induce severe illness, including severe acute respiratory syndrome coronavirus (SARS-CoV), Middle East respiratory syndrome coronavirus (MERS-CoV), Hendra virus, Ebola virus, Marburg virus, and SARS-CoV-2 [1,2,3,4]. Among the *Rousettus* spp., the rousette bat is thought to be a natural reservoir of Ebola and Marburg viruses (family *Filoviridae*) and is a source of virus spillover into human populations; these viruses have frequently caused outbreaks in African countries [5,6]. These days, betacoronavirus genome [7,8] and CoV RNA-dependent RNA polymerase gene [9] were also detected in *Rousettus* spp. The susceptibility of *Rousettus aegyptiacus* to SARS-CoV-2 infection was tested, and it was revealed that 78% of the inoculated bats had a transient infection with detection of SARS-CoV-2 RNA in their lung, lung-associated lymphatic tissue, and trachea [10]. The infected fruit bats were also able to spread the virus to contact bats leading to infection [10]. IgG against Ebola virus and Reston virus was detected in *Rousette aegyptiacs, R. amplexicaudatus*, and *R. leschenaultii* [11,12,13]. In an experiment involving inoculation of *R. aegyptiacs* with Marburg virus, viremia was detected, and a systemic infection developed without any symptoms [14].

Pteropine orthoreovirus (PRV) was a causative agent of respiratory disease in Humans. PRV was first isolated in 1968 from *Pteropus poliocephalus* [15], and then, PRV has been detected in patients with respiratory illness and isolated from several species of flying foxes including *R. amplexicaudatus* and *R. leschenaultii* in South East Asia and Australia [16,17,18,19,20]. Thus, rousette bats are a natural reservoir for a variety of pathogens that cause serious diseases in humans. However, there are limitations in using individual bats to analyze the virus tolerance mechanism in the body in detail.

Knowledge of the bat-specific immune response against virus infection at the organ level has been restricted by the limitations of the in vitro system. Three-dimensional culture systems, such as organoids, have been established and used to study disease modeling, drug testing, stem cell behavior, and epithelial responses to injury and host–pathogen interactions in humans and mice [21,22,23,24,25,26,27,28].

The intestinal tract is known to be one of the gateways for pathogens to enter and exit the body, along with the lungs, and expression of angiotensin-converting enzyme II (ACE2), the receptor of SARS-CoV and SARS-CoV-2, was identified within intestinal tissue with the same level as lung in Egyptian rousette bat [29]. In bats, intestinal organoids from *Rhinolophu sinicus*, a natural reservoir of SARS-CoV, were established, and it was shown that the organoids recapitulated the bat intestinal epithelium [30]. These horseshoe bat enteroids express ACE2 and transmembrane cellular protease serine 2 (TMPRSS2). The bat enteroids developed an cytopathic effect (CPE), and increased viral load was observed until 72 h post-infection. However, the investigators could not consistently sustain long-term expansion, as in human enteroids, using their medium cocktail.

Novel in vitro experimental systems are necessary to analyze virus–host interactions in fruit bats against Ebola and Marburg viruses and to isolate novel viruses from fruit bats. Therefore, we aimed to establish and characterize intestinal organoids from *R. leschenaultii*, assess long-term culture methods, and evaluate their usefulness for studying host–virus interactions.

## 2. Results

### 2.1. Establishment of Intestinal Organoid from Rousettus leschenaultii

**Figure 1:**
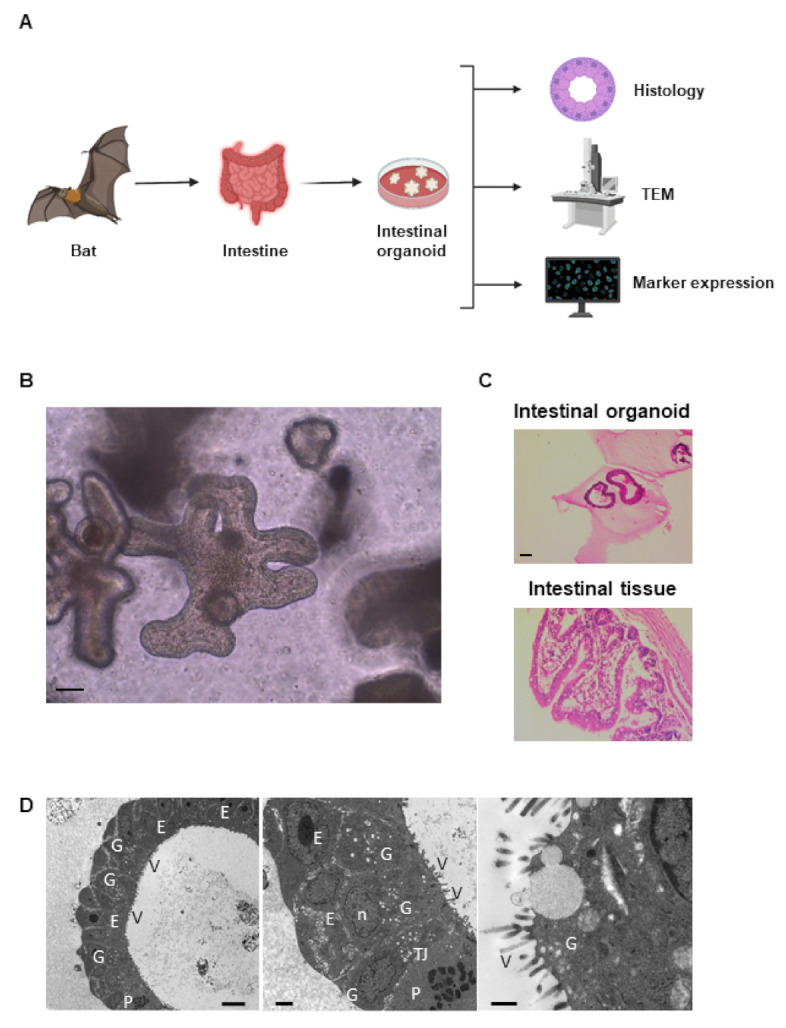
Generation of primary rousette bat intestinal organoids. Experimental schema of establishment and analysis of Rousette bat intestinal organoids (**A**). Bat intestinal tissues were isolated and cultured for generating their organoids. Then, each tissue-derived organoid was used for the analysis of histology, microstructure by transmission electron microscopy (TEM), and marker expression. Representative phase-contrast images of the organoids (passage 8 at day 10). Scale bar: 200 µm (**B**). Hematoxylin and eosin-stained bat organoids and original tissues. Scale bar: 100 µm (**C**). TEM photomicrographs of organoids (**D**). The absorptive epithelial cells (E), nucleus (n), microvilli (V), tight junction (TJ), goblet cells (G), and Paneth cells (P) are shown. Scale bar: 8 µm, 2 µm, 600 nm from left to right panel, respectively.

Rousette bat intestinal organoids were successfully generated and analysed from *R. leschenaultii* that died of natural causes at the zoo and were collected Figure 1A. The structure of the organoid mimicked the multicellular structure of the bat small intestinal epithelium (Figure 1B), and the organoids recapitulated the histology of the original tissue (Figure 1C). In TEM analysis, the ultrastructural morphology of organoids mimicked that of intestinal tissues. The absorptive epithelial cells with characteristics of microvilli, goblet cells, and Paneth cells were identified in the organoids (Figure 1D).

### 2.2. Characterization of Bat Organoid

**Figure 2:**
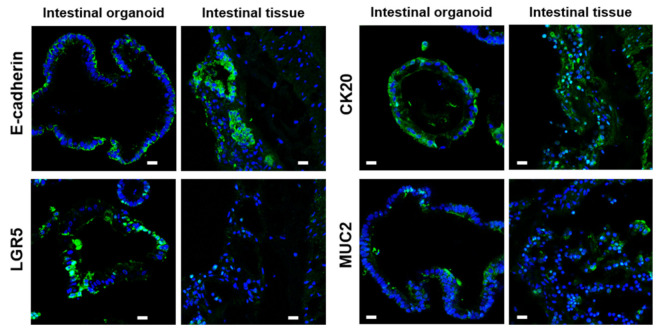
Characterization of the cellular components of the intestinal organoids from rousette bats. Expression of an epithelial cell marker, E-cadherin, an intestinal stem cell marker, LGR5, a mature enterocyte marker, CK20, and an intestinal goblet cell marker, MUC2. Scale bar: 50 μm.

To characterize rousette bat intestinal organoids, the expression of several specific cellular markers of intestinal tissues was evaluated by immunofluorescence staining. E-cadherin, an epithelial cell marker, CK20, a mature enterocyte marker, and MUC2, an intestinal goblet cell marker, were detected in the organoids and bat intestinal tissues, and both E-cadherin and CK20 were positive only in the outer layer of the organoids, whereas LGR5, an intestinal stem cell marker, was more abundant in the organoids than in the original tissues (Figure 2). These data indicate that the organoids recapitulated the cellular components of intestinal tissues with high stemness.

### 2.3. Evaluation of Appropriate Growth and Maintenance Media for Rousette Bat Organoids

**Figure 3:**
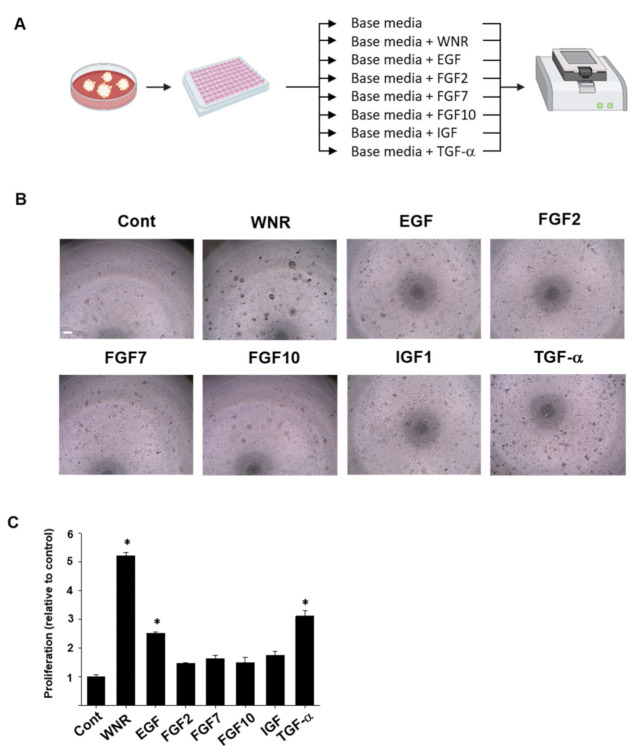
Identification of suitable culture supplements for intestinal organoids from the rousette bat. Experimental schema of identifying suitable media components of rousette bat organoids (**A**). Phase-contrast images of the growth of rousette bat intestinal organoids cultured in base medium alone (Cont) or with different culture supplements. WNR indicates Wnt 3a, Noggin, and R-spondin. Scale bar: 500 µm (**B**). Cell proliferation ratio of organoids in each culture media (**C**). The results were shown as a fold increase relative to control and expressed as mean ± S.E.M. * *p* < 0.05 vs. control.

For long-term culture, the organoids were cultured with several supplements: Wnt 3a, Noggin, R-spondin (WNR), EGF, FGF2, FGF7, FGF10, IGF, or TGF-α. The same number of organoid cells was seeded, and the growth and proliferation rates of the organoids were evaluated. There were significant differences in the effects of supplements on the growth and proliferation rates of rousette bat organoids (Figure 3). On day 7, the growth and proliferation rates were significantly higher when using the base media (Cont) with WNR, TGF-α, or EGF (Figure 3B,C). However, FGF2, FGF7, FGF10, and IGF had no significant effects compared to the control. Based on these results, we identified that WNR, EGF, and TGF-α are essential for the culture of rousette bat intestinal organoids. The organoids maintained active proliferation for 10 months. We also confirmed that long-term cryopreserved rousette bat intestinal organoids grew normally, and subsequent analyses were performed based on these culture conditions (basal medium plus L-WNR condition medium (Wnt, Noggin, and R-spondin), human epidermal growth factor (EGF, 50 ng/mL), and transforming growth factor-alpha (TGF-α, 20 ng/mL).

### 2.4. Susceptibility of the Organoids to SARS-CoV-2 and PRV

**Figure 4:**
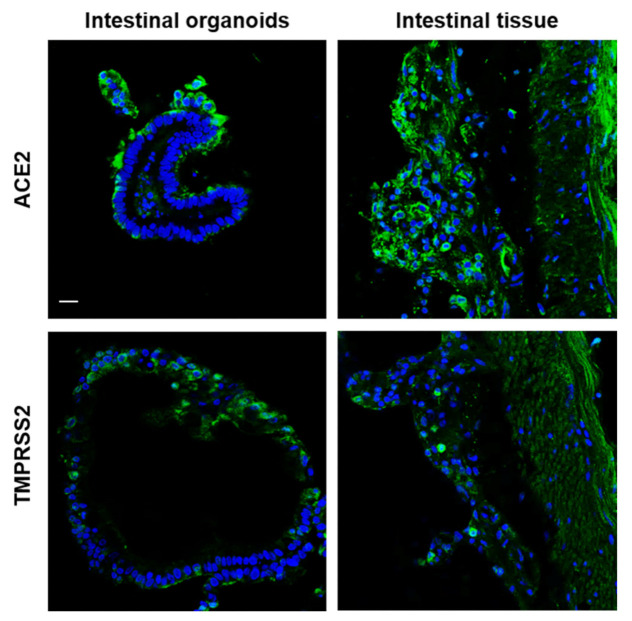
Localization of ACE2 and TMPRSS2 proteins in the rousette bat organoids and the bat intestine. ACE2 (green) or TMPRSS2 (green) proteins were stained using antibodies against each human homologue and merged with DAPI (blue). Scale bar: 50 μm.

**Figure 5:**
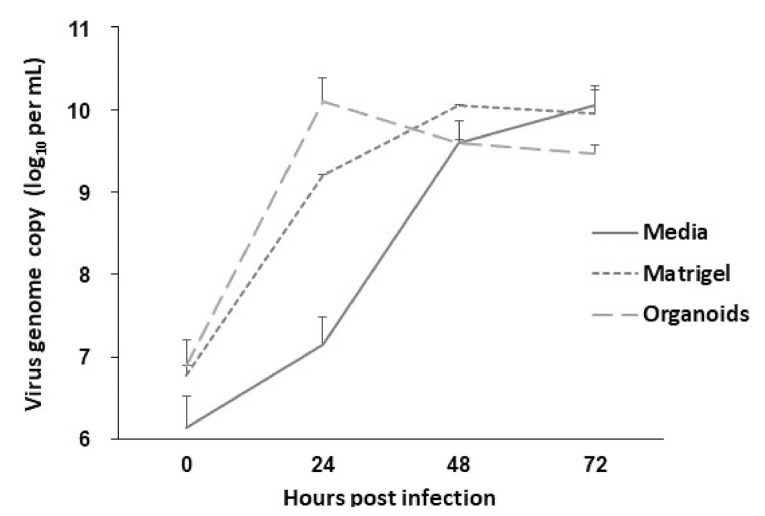
Growth curve of PRV in Matrigel, culture media, and organoids over 72 h. Rousette bat intestinal organoids were infected at MOI = 0.1. The amount of PRV genome was quantified by RT-PCR assay. The infection experiment was performed in technical triplicate, and the quantification of the virus genome was performed in duplicate. The values were shown as the mean ± S.E.M (*n* = 3).

**Figure 6:**
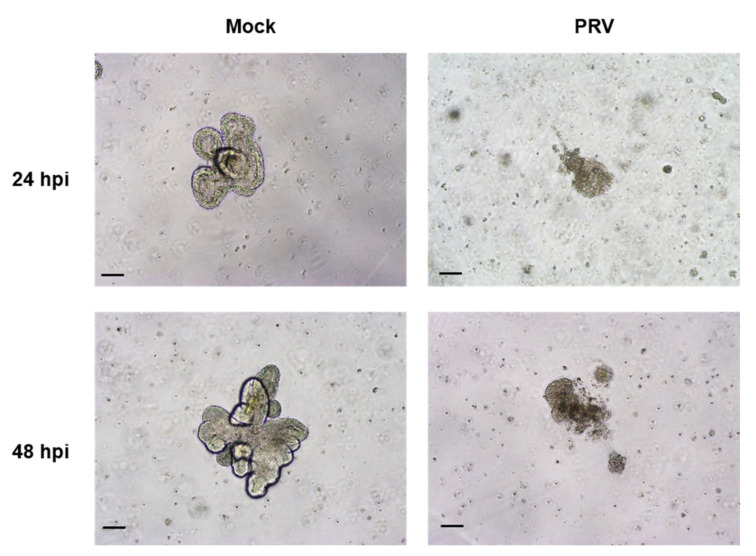
Representative phase-contrast images of mock-infected (Mock) and PRV-infected organoids (PRV). The upper and lower were 24 and 48 hpi, respectively. Scale bar: 200 µm.

It has been confirmed that SARS-CoV-2 uses the ACE2 receptor for cell entry [4] with the aid of TMPRSS2 [31]. We analyzed the expression of ACE2 and TMPRSS2 in bat intestinal organoids. ACE2 was clearly expressed on the apical surface or basal membrane of organoids and in the intestinal tissues (Figure 4). TMPRSS2 was expressed not only at the basal surface but also at several sites in organoids and their original tissues (Figure 4). The amplification of SARS-CoV-2 could not be observed in either Matrigel or culture media of intestinal organoids from *R. leschenalutii*. The rousette bat intestinal organoid did not show CPE, and the virus genome was not detected at any time point (data not shown). In contrast, PRV was amplified in the rousette bat intestinal organoids (Figure 5). The levels of PRV genome in Matrigel increased to 3.2 × 10^9^ copies/mL at 24 hpi and peaked at 1.4 × 10^10^ copies/mL at 48 hpi. In the culture media, the levels of the PRV genome were lower than those in Matrigel. At 24 hpi, the levels of the PRV genome were 1.4 × 10^6^ copies/mL and peaked at 1.1 × 10^9^ copies/mL at 72 hpi. CPE was identified at 24 hpi (Figure 5). In mock infection, the three-dimensional structure for the intestinal organoids at 24 and 48 hpi was maintained. On the other hand, PRV-infected organoids showed a progressive cytopathic effect indicated by a disruption of structural integrity as loss of membrane and cell–cell attachments, appearance of multinucleated giant cell-like structure, and apoptosis-like changes (Figure 6). These changes indicate that the rousette bat intestinal organoids are susceptible to PRV but not to SARS-CoV-2.

## 3. Discussion

The bat (order *Chiroptera*) is a natural reservoir of several emerging and re-emerging viruses that induce severe infectious diseases, including Ebola virus, Marburg virus, Nipah virus, Hendra virus, SARS-CoV, MERS-CoV, and SARS-CoV-2. To investigate these bat-derived infectious diseases, in vivo bat assays are an important experimental tool. However, bats are wild animals, not experimental animals, and it is very difficult to conduct reproducible animal experiments. In this study, we successfully developed intestinal organoids from a Megachiroptera, *Rousettus leschenaultia*, and established a long-term stable culture method for the first time. The rousette bat intestinal organoids showed the same cellular composition as intestinal tissues from morphological and immunohistochemical characterizing analyses, as demonstrated previously in bats [30] and humans [32].

Additionally, we identified the appropriate culture supplements for maintaining the long-term culture of rousette bat intestinal organoids without ceasing active proliferation. However, for intestinal organoids from the horseshoe bat *Rhinolophus sinicus*, a long-term stable culture could not be achieved [30]. In rousette bat intestinal organoids, the base medium with WNR, TGF-α, and EGF showed promising effects on growth and proliferation (Figure 3). In intestinal organoid cultures from other animals, including humans and mice, the combination of EGF and WNR is necessary to indefinitely proliferate and expand cells and recapitulate the differentiated cell diversity of the original tissue [33] because these supplements are necessary for the survival of LGR5^+^ intestinal stem cells [34,35]. Although IGF exists at a high level in the human bloodstream [36] and has been demonstrated to stimulate crypt expansion in mice [37,38], it did not promote the proliferation of the organoid. In the same context, FGF2, which is expressed in the mesenchymal cells adjoining the intestinal crypts of mice and is upregulated following tissue injury to promote regeneration [39], had no significant effects on organoid expansion. FGF7 was identified as a differentiation factor in the developing lung [40] and controls the branching of the airway epithelium by promoting epithelial cell expansion and proliferation [41,42]. Lung organotypic culture studies revealed that the addition of FGF7 to the culture medium can induce AT2-like epithelial cell differentiation, even in the absence of any mesenchyme or serum [42,43]. TGF-α, a ligand for the EGF receptor, is expressed in many types of cells, including intestinal and lung cells, and shows a promising effect on their growth and proliferation for maintaining their long-term culture. TGF-α binds to the EGF receptor and activates tyrosine kinase signaling, which results in the proliferation and differentiation of gastrointestinal and lung epithelium, especially following injury [44,45,46]. In addition, CD24^+^ Paneth cells express EGF, TGF-α, and Wnt3a, which are necessary for stem cell maintenance in the culture of intestinal crypts [47]. In the lung, EGF and TGF-α mediate morphogenesis and repair [44]. EGF has also been detected in AT2 cells and bronchiolar, ciliated, and non-ciliated cells [48]. EGF and TGF-α have been reported to alter branching morphogenesis and differentiation of the developing lungs of mice [49]. These data indicate the importance of these supplements in the culture medium of bat rousette intestinal organoids.

The rousette bat intestinal organoid is a useful tool for the evaluation of virus susceptibility in vitro. In this study, we showed that SARS-CoV-2 was not amplified in the rousette bat intestinal organoids, but PRV increased 24 h post-inoculation. The sequence of ACE2 is conserved between *R. aegyptiacus* and *R. leschenaultii*, and ACE2 of *R. aegyptiacus* has the ability to support SARS-CoV-2 entry [50]. In binding assay between ACE2 orthologs of animals including *R. leschenaultia* and the receptor-binding domain for SARS-CoV-2 and RaTG13, closer species of SARS-CoV-2 detected from Rhinolophus bats, the affinity of ACE2 to RBD of each virus species was different among species in bats, and ACE2 of *R. leschenaultia* showed a low affinity [51]. Experimentally, intranasal inoculation of SARS-CoV-2 with *R. aegyptiacus* resulted in a transient infection of the respiratory tract [10]. However, WINV1-CoV and SARS-like CoV isolated from *Rhinolophus sinicus* did not cause a robust infection in *R. aegyptiacus*, and the cell line from the intestine of *R. leschenaultii* did not support SARS-CoV-2 infection [29,50]. Furthermore, the susceptibility of rousette bats to SARS-CoV-2 is controversial. In this study, the bat intestinal organoids did not show susceptibility to SARS-CoV-2, although ACE2 and TMPRSS2 were expressed. The intestinal organoids from *Rhinorophus sinicus*, one of the *Rhinolophus* spp. thought to be a natural reservoir of SARS-CoV and SARS-CoV-2, were susceptible to SARS-CoV-2 infection and showed viral replication within 72 h post-infection with MOI = 0.1 [30]. PRV was also isolated from patients with respiratory tract infections and has been isolated from bats, including *R. leschenaultii* [15,18,52,53], and the PRV Samal-24 strain was isolated from *E.* *spelaea* in the Philippines in 2013 [20]. PRV could be propagated in the rousette bat intestinal organoid and showed CPE at 48 h post-inoculation in this study. These results indicate that rousette bat intestinal organoids are useful tools to evaluate virus susceptibility and tolerance mechanisms of viruses, including Ebola and Marburg viruses.

## 4. Materials and Methods

### 4.1. Preparation of Rousette Bat Tissues

Intestinal tissue was collected from five rousettes of Leschenault (*Rousettus leschenaultii*), which were kindly supplied by Ueno Zoo (Tokyo, Japan) and Chiba Zoological Park (Chiba, Japan). These bats were confirmed to have died of natural causes at the zoo, and intestinal tissues were collected. The collected tissues were placed in a preservation culture medium and transported to the laboratory under refrigeration conditions.

### 4.2. Establishment of Bat Intestinal Organoid

The collected intestinal tissues were washed three times with cold phosphate buffer saline (PBS), chopped into fine pieces, and transferred into tubes containing advanced Dulbecco’s modified Eagle’s medium (DMEM) with 0.125 mg/mL Liberase TH for digestion and isolation of intestinal crypt cells. The tubes were then incubated at 37 °C in a shaking incubator for 30 min and pipetted every 15 min. The resultant cell suspension was then passed into a 70 µm nylon net cell strainer and spun down at 600× *g* for 5 min at 4 °C. The cell pellets were then washed three times with PBS, suspended in Matrigel on ice, and dispersed in a 24-well plate (40 μL/well). After the Matrigel was polymerized to form a droplet in a CO_2_ incubator at 37 °C for 30 min, 500 µL of stem cell-stimulating medium was added to each well. The medium was changed 2–3 times weekly. Organoids were passaged every 7–14 days using 5 mM EDTA-PBS solution at 1:2–4 split as described previously [54]. Photomicrographs of the organoids were acquired using an Olympus CKX-31 inverted microscope (Olympus, Tokyo, Japan).

### 4.3. Investigation of Suitable Supplementation for Organoid Culture

To investigate the suitable culture conditions for rousette bat organoids, various supplements were individually applied, and the growth, proliferation rate, and maintenance of the organoids were evaluated. The organoids were treated with TrypLE (Life Technologies Co., Grand Island, NY, USA) for 5 min and strained using a 70 µm cell strainer (Falcon, Cary, NC, USA). Thereafter, 1 × 10^3^ organoid cells were seeded into 10 µL Matrigel in 96-well culture plates and incubated at 5% CO_2_ at 37 °C for 30 min. In the culture of intestinal organoids from humans, mice, and other animals, human epidermal growth factor (EGF) and WNR (Wnt, Noggin, and R-spondin) were shown to be necessary for the proliferation, expansion of the cells, and maintaining the cellular diversity of the original tissues [33,34,35]. Thus, we tried the effects of several culture supplements on the growth and proliferation of organoids. After polymerization of the Matrigel, the organoid cells were cultured under conditions in which one of the following supplements was added to the basal medium and compared to basal medium only: L-WNR condition medium, EGF (50 ng/mL, PeproTech, Inc., East Windsor, NJ, USA), fibroblast growth factor-2 (FGF2, 20 ng/mL, PeproTech, Inc.), FGF7 (5 ng/mL, PeproTech, Inc.), FGF10 (20 ng/mL, PeproTech, Inc.), insulin-like growth factor (IGF, 10 ng/mL, PeproTech, Inc.), and transforming growth factor-alpha (TGF-α) (20 ng/mL, PeproTech, Inc.). Quantification of the effects on growth and proliferation of organoids was performed on day seven after culture using the PrestoBlue assay kit (Thermo Fisher Scientific, Eugene, OR, USA) and a microplate reader (TECAN, Seestrasse, Switzerland).

### 4.4. Histopathological and Immunohistochemical Examination

Hematoxylin and eosin staining was conducted for histopathological analysis, as described previously [25,55,56]. Concisely, after fixing the intestinal tissue and organoids with a 4% paraformaldehyde (PFA) solution (Fujifilm, Osaka, Japan) at room temperature (RT) for 24 h and 2–3 h, respectively, these samples were embedded in paraffin and sliced into 5 μm-thick sections and mounted onto MS-coated glass slides. Following deparaffinization, the sections were stained with hematoxylin and eosin using a standard protocol, and the photomicrographs were captured using an Olympus BX-52 inverted microscope. Immunofluorescence staining of intestinal tissues and organoids was performed as previously described [54,55]. The tissues and organoids were fixed in 4% PFA for 1 h at RT and dehydrated with a 30% sucrose solution at 4 °C overnight. Subsequently, they were embedded in OCT compound (Sakura Finetek Inc., Torrance, CA, USA) and kept at −80 °C until slicing. The frozen sections were prepared and treated with 1.5% normal goat serum in PBS at RT for 1 h. Sections were then incubated with primary antibodies (E-cadherin; 1:100, cytokeratin 20 (CK20); 1:200, mucin 5AC (MUC5AC); 1:200, transmembrane protease serine 2 (TMPRSS2); 1:200, CK5; 1:200; angiotensin-converting enzyme II (ACE2); 1:200, mucin 2 (MUC2); 1:200, leucine-rich repeat-containing G-protein coupled receptor 5 (LGR5); 1:200, secretoglobin family 1A member 1 (SCGB1A1); 1:200, and surfactant-associated protein C (SFTPC) 1:200) and kept at 4 °C overnight. The sections were then washed three times with PBS and incubated with the secondary antibodies and Hoechst stain for 1 h in the dark at RT. After washing, a small drop of Fluoromount solution (Diagnostic Biosystems, Pleasanton, CA, USA) was added per section and covered by a coverslip. The sections were then allowed to dry for 2 h in the dark at RT. Images were captured using a confocal microscope (LSM 800; ZEISS, Copenhagen, Germany).

The antibodies used in this study were as follows: E-cadherin (R&D System, Minneapolis, MN, USA); CK20; MUC5AC; TMPRSS2; SCGB1A1 (Bioss, Woburn, MA, USA); CK5, ACE2; MUC2 (GeneTex, Inc., Irvine, CA, USA); LGR5; (Abgent California, CA, USA); SFTPC (Affinity Biosciences LTD, OH, USA). Fluorescent secondary antibodies were as follows: Alexa Fluor 488 goat anti-rabbit IgG and Alexa Fluor 488 goat anti-mouse IgG (Thermo Fisher Scientific Inc.).

### 4.5. Transmission Electron Microscopy (TEM)

After the organoids were fixed with 2.5% glutaraldehyde for 3 h at RT in 0.1% cacodylate (pH 7.4), they were washed once with 0.1 M cacodylate (pH 7.4), followed by incubation in 2% osmium tetroxide and 1.5% K_4_Fe(CN)_6_ in 0.1 M sodium cacodylate (pH 7.4) for 2 h at 4 °C. After rinsing with distilled water, the organoids were dehydrated in a graded ethanol series (50%, 70%, 80%, 90%, 95%, and 99.5 up to 100%) and embedded in Epon. Ultrathin sections of 70–110 nm were cut with a diamond knife on a Leica UC7 ultramicrotome and transferred onto 50-mesh copper grids covered with a form bar and carbon film. The sections were post-stained with uranyl acetate for 15 min at RT and lead citrate. Sections were imaged using a transmission electron microscope (H-7500, Hitachi, Tokyo, Japan) using a TEM digital camera (NanoSprint500, Hitachi).

### 4.6. Sensitivity of Organoid to SARS-CoV-2 and Pteropine Orthoreovirus

The Pteropine orthoreovirus Samal-24 strain (PRV) was kindly provided by M. Shimojima, Department of Virology I, NIID, Japan, and SARS-CoV-2 was isolated from swab samples at NIID, Japan. To investigate the replication kinetics of these viruses in the organoid, the organoid was infected with the virus at an MOI of 0.1 [30]. To prepare the organoids for virus infection, 500 μL of ice-cold 5 mM EDTA-PBS was added per well of a 24-well plate and incubated on ice for 90 min. Thereafter, organoids were collected, trypsinized, and passed through a 70 μm cell strainer. An equal number of cells was seeded per well. After enough growth and typical morphology, the Matrigels with organoids were sheared gently and organoids were collected. After centrifugation, equal volumes of organoid pellets were confirmed using a weight/volume-based approach, and MOI 0.1 was then adjusted. Subsequently, organoids were incubated in a medium containing MOI 0.1 of SARS-CoV-2 or PRV at 37 °C for 1 h. After incubation, the organoids were washed once with PBS, resuspended in Matrigel on ice, and dispersed in a 24-well plate (40 μL/well). After the Matrigel was polymerized to form a droplet in a CO_2_ incubator at 37 °C for 30 min, 500 µL of medium was added to each well. At 2, 24, 48, and 72 h post-inoculation (hpi), media was collected, the Matrigel was washed once by ice-cold PBS, and 350 μL of 5 mM EDTA-PBS was added to each well and incubated on ice for 90 min. The dissociated Matrigel was centrifuged, and the supernatant solution was collected and used for RNA extraction with a High Pure Viral RNA kit (Roche) for RNA extraction. Extracted RNAs were used to detect each viral genome by RT-qPCR, as described previously [57,58]. Matrigel was also collected to evaluate the viral genome level using a real-time PCR assay. Infection experiments and genome detection were performed in triplicate and duplicate, respectively.

### 4.7. Statistical Analysis

Data are expressed as the mean ± SEM. Statistical significance was evaluated using one-way analysis of variance (ANOVA) followed by Bonferroni’s test. Statistical significance was set at *p* < 0.05.

## 5. Conclusions

We have improved the culture method of rousette bat intestinal organoids for long-term use, and the intestinal organoids were able to reproduce the multicellular structure of the intestinal epithelium. We expect that the long-term storage of rousette bat intestinal organoids produced in this study will provide a new experimental platform for the isolation and study of other bat pathogens with high zoonotic potential.

## Data Availability

The detailed data of the current study are available from the corresponding authors on reasonable requests.
